# Selective D3 Receptor Antagonist SB-277011-A Potentiates the Effect of Cocaine on Extracellular Dopamine in the Nucleus Accumbens: a Dual Core-Shell Voltammetry Study in Anesthetized Rats

**DOI:** 10.3390/s8116936

**Published:** 2008-11-04

**Authors:** Francesco Congestri, Francesca Formenti, Viviana Sonntag, Gael Hdou, Francesco Crespi

**Affiliations:** Biology Dept, GlaxoSmithKline, Medicines Research Centre, Verona, Italy

**Keywords:** *In vivo* voltammetry, rat brain, nucleus accumbens, core, shell, Dopamine, D3 receptor antagonist

## Abstract

Dopamine (DA) D3 receptors have been associated with drug intake and abuse and selectively distribute in the brain circuits responding to drug administration. Here we examined the effects of an acute systemic administration of cocaine (15 mg/kg) alone or preceded by treatment with the selective D3 receptor antagonist SB-277011-A (10 mg/kg) on DA levels concurrently in the rat nucleus accumbens shell and core sub-regions (NAcshell and NAccore, respectively). It is shown that cocaine increases extracellular DA in both compartments and that blocking D3 receptors with SB-277011-A, although the latter is devoid of dopaminergic effects *per se*, potentiates these effects. No differences in the amplitude of the response were observed between NAcshell and NAccore compartments, though the dopaminergic response in the NAcshell was transient whereas that in the NAccore rose slowly to reach a plateau. These results demonstrate the feasibility to use multiprobe voltammetry to measure discrete monoaminergic responses in discrete areas of the brain and confirm the effect of D3 receptors antagonist at modifying the neurochemical effects of cocaine.

## 1. Introduction

### 1.1. NAc shell and core

Over the past decades, a large number of studies have underlined the role that the dopaminergic mesocorticolimbic system sustains in neurochemical and behavioural responses to drug use and abuse [1]. The mesocorticolimbic system is constituted by the dopaminergic neurons located in the ventral tegmental area (VTA) that mainly project to the subcortical nucleus accumbens (NAc) and cortical structures such as the medial prefrontal and motor cortices [2]. Neuroanatomical, neurochemical and neurobehavioral specializations have led to the subdivision of NAc into an external shell and internal core regions. The division of the NAc into core and shell is based on morphological differences [3] as well as in the afferent and efferent connections of the two regions [4, 5].

In particular, the shell establishes intense connections with the limbic system and the hypothalamus and is particularly responsive to the reinforcing properties of addictive compounds, [6]. Differences in the distribution of various chemical messengers such as substance P, dopamine (DA), and enkephalin have been also described between the two areas [7].

In the early 90s DA metabolism was shown to be increased in the shell but not in core following mild stress. In contrast, antipsychotic drugs such as haloperidol were found to increase DA metabolism more in the core than in the shell, whereas clozapine produced approximately the same effect on DA metabolism in both subregions. Based on such findings, it was concluded that the shell relates to mesolimbic pathway involved largely with emotional and motivational processes, while the core is functionally related to the striatal area and plays a role predominantly in motor functions [8, 9].

In addition, Cadoni *et al.* [10] have demonstrated differential neurochemical and behavioural response across the sub-territories of the NAc following application of cannabinoids. Furthermore, it has been shown that local injection of mu or delta opioid agonists into the shell but not into the core increased intracranial self stimulation (ICSS) [11], yet when DA agonists were injected into the shell, no behavioural activations were observed, while they were obtained following local injection into the core [12]. Besides, recent studies have highlighted the function of the NAcshell in mediating the appetitive and aversive feature of environmental stimuli that takes part, to some extent, in the emotional component of stimuli [13]. In contrast, the NAccore has been implicated in motor-related behaviours associated with psychostimulant administration as well as the expression of withdrawal symptoms after termination of repeated heroin treatment [14].

Altogether, the data mentioned support the indication that the accumbens core is densely linked to the neostriatum in terms of input, structure and contribution in motor function. In contrast, the shell is linked with limbic and subcortical areas, is considered to be a component of the ‘extended amygdala’ and to play a role in motivation and emotional responding [15].

In particular, most of the *in vivo* data cited above have been obtained with the microdialysis methodology. Likewise, very few studies in NAccore and shell have been reported up to date with the other major *in vivo* electrochemical methodology i.e. voltammetry. For instance, a very short lasting (seconds) increase of the catecholaminergic related voltammetric signal in shell but not in core in rats given free-choice access to a novel environment has been described [16]. On the other hand, a more recent voltammetric report indicates that DA neurons innervating the core or the shell appear to be involved distinctly within a conditioned paradigm to an olfactory stimulus, depending on the aversive or the attractive value of it, respectively [17].

### 1.2. DA receptors in NAc

The NAc contains high density of the D2-like family of dopamine receptors to which belong the D3 receptors. To a lesser extent, D3 receptors are expressed in the amygdala, [18] and much less expressed in the dorsal striatum. Their specific distribution among other dopamine receptors family confers upon them a potential role in those processes associated with cognitive and emotional behaviours [19-21] as well as drug abuse and reinforcement [22].

Under physiologic conditions the extrasynaptically located DA transporter (DAT) affects extrasynaptic DA levels helping terminate DA action and favouring its recycling [23]. Cocaine binds with low affinity to DAT (pKi = 6.38 [24]) as well as noradrenalin and serotonin transporters. Cocaine binding at these transporters blocks the recapture mechanism of the respective neurotransmitters leading, in the case of DAT, to increased DA levels in the synaptic cleft and therefore in the extracellular space where the electrode is measuring.

SB-277011-A is a selective D3 receptor antagonist with high affinity for human (pKi 7.95) and rat (pKi 7.97) cloned DA D3 receptor, with an *in vitro* D3/D2 affinity ratio for human and rat of 120 and 80, respectively and with a 100-fold selectivity over 66 other receptors, enzymes, and ion channels [25].

The compound has a good brain penetration (cerebral to blood ratio of 3.6:1 [26] suggesting that its action is mainly mediated by central antagonist at the D3 receptor). SB-277011-A was shown to inhibit cocaine and nicotine self administration and reduce craving for the drugs after withdrawal. Furthermore SB-277011-A was shown to reduce cue-, stress- and drug-induced relapse in cocaine self administration models and to reduce cocaine conditioned place preference [27-29]. These behaviours have been associated with modulation of monoamines dynamics in the NAc [18].

Hence the present work aimed at examining the effects of the D3 receptor antagonist SB-277011-A on DA dynamics in the NAc core and shell subterritories *per se* and preceding acute cocaine administration. We used a dual *in vivo* voltammetric probe approach in anesthetized male adult rats to monitor extracellular levels of DA in both the NAccore and NAcshell sub-regions simultaneously. We show that the increase in DA levels by cocaine was similar in both NAc subregions and that SB-277011-A alone had no effect on DA levels. Finally, pre-treatment with SB-277011-A significantly enhanced the cocaine-increased DA levels in both core and shell with respect to cocaine alone.

## 2. Experimental Section

### 2.1. Subjects

Adult male CD rats (250-310 g) were employed in this study. Rats were subjected to a normal light/dark cycle (light on at 7.00 a.m.) with food and water available *ad libitum*. All procedures were carried out in accordance with the Italian law (Legislative Decree no.116, 27 January 1992), which acknowledges the European Directive 86/609/EEC, and were fully compliant with GlaxoSmithKline policy on the care and use of laboratory animal and codes of practice.

### 2.2. Drugs and treatment groups

Following basal recording for 30 min, the animals were randomly assigned to one of the three treatment groups. Animals from group 1 received systemic (intraperitoneally, i.p.) administration of vehicle (cavasol 10% in water, 2mL/kg, n=4, first injection at time 30 min, second injection at time 120 min), group 2 animals received vehicle and cocaine (Sigma-Aldrich, 15 mg/kg i.p., n=3) and animals from group 3 received SB-277011A (GlaxoSmithKline, 10 mg/kg i.p., followed 90-100min later by cocaine (10 mg/kg i.p., n=4). SB-277011A and cocaine were both dissolved in cavasol 10%.

### 2.3. Voltammetry and carbon fibre microelectrodes (micro-biosensors)

**Figure 1.**
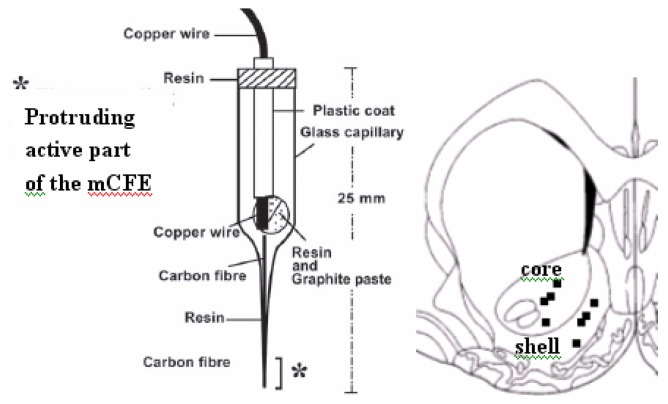
LEFT: schematic representation of the carbon fibre microelectrode used in the present experiments. RIGHT: Example of locations of the amperometric recording sites in core and shell indicated by the black squares drawn onto a representative section of the rat brain: coordinates accordingly to Paxinos and Watson [34], see Experimental Section.

Voltammetry was applied by means of a μAutolab polarograph (EcoChemie, The Netherlands) linked to an IBM PC computer equipped with a General-Purpose Electrochemical System Software (GPES) package. Voltammetric measurement was performed with a three-electrode potentiostat system made of a silver/silver chloride (Ag/AgCl) reference electrode, a copper wire auxiliary (counter) electrode both approximately 100 μm in diameter [30, 31], and a 30 μm diameter carbon fibre microelectrode (mCFE) constituting the working electrode that was electrically treated and coated with the perfluorinated ion-exchange resin Nafion to lower sensitivity for the interfering anion metabolite DOPAC as previously described [32, 33]. This treatment was shown to increase sensitivity, selectivity and reliability of the micro-sensor to quantification of biogenic amines. A 70 Hz triangular waveform was applied in three stages, 0 to +2.4 V for 8 sec; 0 to +2.0 V and 0 to +1.8 V for 10 sec each. Two successive continuous potentials were subsequently applied to the mCFE: +1.0 V and -0.6 V, 4 sec each. This electrochemical treatment was carried out with the auxiliary, reference and working electrodes immersed in 0.01 M phosphate-buffered saline (PBS) at pH 7.4. Such treatment affects the active tip of the carbon fibre (working electrode) that protrudes from the end of the glass pipette (30 μm diameter, 500 μm length) (Figure 1). Then, for in vivo studies, the choice of Nafion mCFE [32] upon NA-CRO mCFE [33] rose from the evidence of a similar sensitivity of both type of sensors when used with the amperometric method. Similarly, in vitro tests showed that the Nafion mCFE appeared to be insensitive to DOPAC at micromolar concentration [32].

### 2.4. In vivo voltammetry in dual-probe preparation

Rats were anaesthetised with urethane (1.5 g/kg i.p.) and held in a Kopf stereotaxic frame throughout the experiment. Two mCFE were inserted with their active tips in core and shell of the NAc, respectively. The “working” electrodes (mCFE) were placed side by side in a dedicated holder. Their active tips were inserted in each animal ipsilaterally in the cerebral areas studied as calculated from bregma according to Paxinos and Watson [34]: NAccore: antero-posterior (AP) = 1.6; medio-lateral (ML) = 1.6; dorso-ventral (DV) =7.2 mm; NAcshell: AP: 1.6; ML: 0.8; DV: 7.8 mm. The reference and auxiliary electrodes were positioned between the bone and the *dura mater* through two adjacent holes (200 μm diameter) that were drilled in the parietal bone.

The amperometric parameters were selected to achieve real time measurement of DA-like oxidation current *in vivo* based upon *in vitro* calibration that was performed with Differential Pulse Voltammetry (DPV) associated with Nafion mCFE in solution.

**Figure 2.**
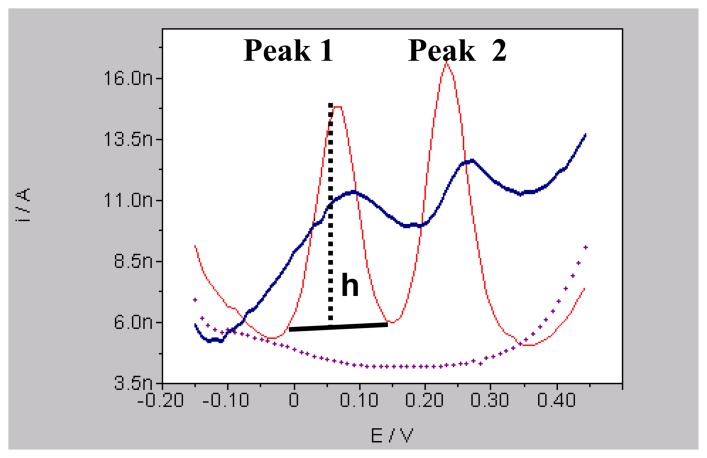
*In vitro* and *in vivo* differential pulse voltammograms obtained with mCFE. Dotted line shows signals obtained in phosphate buffer solution (PBS, pH 7.4), red line shows signal for DA (Peak 1) and 5-HT (Peak 2) obtained in PBS containing at 100 nM and blue line represents in vivo signals obtained in the NAc of anaesthetised rats.; **h**: height of the peak, measured in nanoAmperes (**I/A**: Intensity of current). This perpendicular line also determined the exact potential value (**E**: espressed in Volts) of each signal on the abscissa.

DPV parameters used were as follows: initial potential (Ei) -100 mV, final potential (Ef) +200 mV, scan step was 50 mV.sec^-1^, scan duration: 6 sec, filter: 0.1 Hz, frequency of scans: every 5 min, filter: 0.1 Hz. The polarograph automatically measured the current (nA) coming from peak oxidation of DA by measuring the perpendicular height (h) from the top of the peak to the tangent line drawn between its shoulders as shown in Figure 2. This perpendicular line also determined the exact potential value (E) of each signal on the abscissa. The oxidation potential for DA was 68 ± 24 mV in the buffer solution (PBS, pH 7.4, 37 °C). Therefore, *in vivo* amperometric analysis of DA was performed at the potential +90 mV: i.e. the voltage of the potentiostat was increased instantaneously to the selected potential that corresponds to the potential of a complete oxidation of DA in our amperometric conditions. DA-related currents in core and shell (in nanoAmperes) were detected in time intervals of 0.2 Sec.

### 2.5 histological analysis of mCFE placement in the NAc

At the end of each experiment, an electrolytic lesion (5 Volts, 5 sec) was made through the carbon fibre electrode for histological verification of the recording site. Then the animal was sacrificed, its brain removed and quickly frozen. Each brain was then sliced on a microtome (50 μm), stained (Nissl), and examined under microscope for electrode placement (see Figure 1).

### 2.6. Data analysis

Each 0.2 sec DA measurement was added and averaged over 5 min bins. Averaged data were transformed in percent of the averaged last 15-min basal levels prior to any treatment. Data have been log transformed and statistically analyzed using a three-way ANOVA with between-subjects factors of treatment and NAc sub-region and repeated measurement factor of time using Statistica, version 8.0. Planned comparison *post-hoc* test was applied on treatment by time interaction since the factor area was not significant per se nor in interaction with all the other factors. Statistical significance was set at p < 0.05 for all tests.

## 3. Results and Discussion

Mesolimbic dopamine (DA) neurotransmission plays a central role in the expression of drug of abuse-seeking behaviour, pharmacological effects of drug intake and relapse to repeated drug taking (for reviews see ref 35 and 36]. Among all DA receptor subtypes, the DA D3 receptor that is highly expressed in brain reward-related regions such as the ventral tegmental area (VTA), NAc and amygdala has been shown to interact with these behaviours; for a review see [18].

Previous studies have shown that systemic administration of SB-277011-A, a brain penetrant and selective D3 receptor antagonist with 100-fold selectivity over the D2 receptor [25] can alter the spontaneous activity of DA neurons in the VTA [37]. Furthermore, this compound was shown to interfere with the rat behaviour that resemble abuse behaviour in humans including self-administration, conditioned place preference, cue-induced and drug-induced relapse [18, 29, 38-40].

Thus, given the close association between DA dynamics in the mesocorticolimbic regions of the brain and behaviours associated with drug intake, the present work aimed at analysing the putative influence of SB-277011-A on DA dynamics in the shell and core of NAc in anaesthetised rats. Hence, we measured its effects on DA levels *per se* as well as when DA levels were enhanced by cocaine administration. In particular, we measured these effects simultaneously in the NAccore and NAcshell by means of a new dual-probe approach Namely, two specifically-treated mCFEs [32] associated to direct current amperometry (DCA) were applied to measure DA-related signals in the two adjacent NAc subregions. This approach allows continuous, real time in situ detection of DA without the need for sample collection, preparation or chromatographic analysis [30].

### 3.1. In vivo dual probe amperometry in core and shell: effect of treatments

The results obtained with the amperometric dual probing in core and shell of NAc show similar basal levels of extracellular DA when substracting background current i.e. 3.9 ± 1.9 nA or 3.4 ± 2.1 nA in the core or shell, respectively. Comparable values were observed for DA peak when monitoring basal levels using DPV (data not shown).

**Figure 3.**
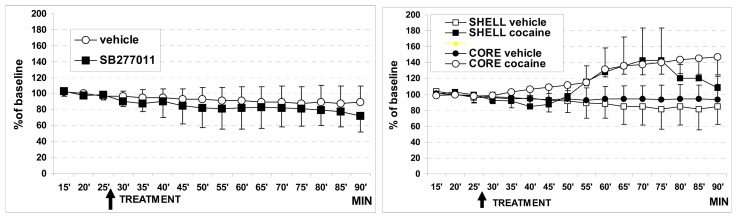
Amperometric analysis of endogenous levels of DA in NAccore and in NAcshell. Data are expressed in percentage of the basal DA values preceding treatment(s). The last 15 min out of 30 min of basal recordings are illustrated: TOP: the arrow indicates the acute i.p. injection of vehicle (cavasol 10 %, open circles, n = 4) or that of SB-277011-A (10 mg/kg, black squares, n = 4). BOTTOM: the arrow indicates acute i.p. injection of vehicle (cavasol 10%, n = 4 each area) OR cocaine (15 mg/kg., n = 3 each area). Data are presented as mean ± SD.

No statistical differences were observed between these areas following two successive vehicle i.p. treatments (cavasol 10 %, group 1), performed at 30 and 120 min of recording period, respectively, thus vehicle data have been plotted to perform the comparison vehicle versus drug treatments. Similarly, treatment with SB-277011-A (10 mg/kg, n = 4) did not alter significantly DA levels in both brain areas studied (see Figure 3 top). In contrast, the present results demonstrate that the acute administration of cocaine (15 mg/kg i.p., group 2) produced a similar, significant increase up to approx. 140 % of DA levels within 40 min in both the core and shell of NAc (Figure 3 bottom). More precisely, the statistical analysis revealed a significant effect of time F_(13,117)_ = 2.6, p = 0.003 and treatment by time interaction F_(13,117)_ = 4.43, p = 0.001 in the NAccore while in the NAcshell it revealed a significant effect of time F_(13,117)_ = 2.12, p = 0.018 and treatment by time interaction F_(13,117)_ = 3.03, p = 0.001. However, no significant difference was observed between shell and core when comparing the effect of cocaine on DA release in both areas. Thus the data from each animal were meaned across regions and values were plotted for comparison versus vehicle and versus SB-277011-A treated rats.

**Figure 4.**
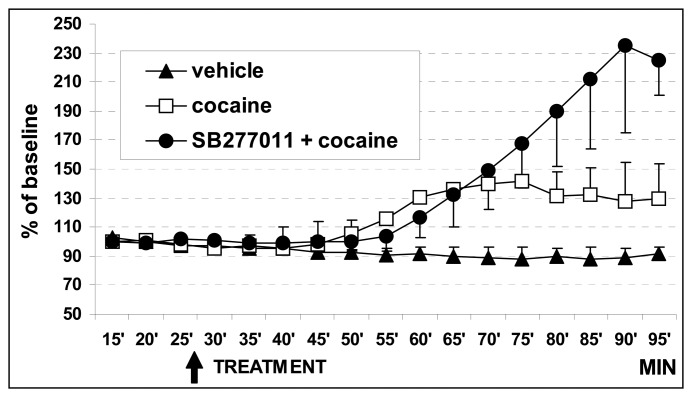
Amperometric analysis of endogenous levels of DA in NAccore and in NAcshell. The last 15 min out of 30 min of basal recordings are illustrated. Data are expressed in percentage of the basal DA values preceding treatment (arrow) i.e. cavasol 10 % (n=4), black triangles, cocaine 15 mg/kg. open squares (n= 3) or SB-277011-A 10 mg/kg + cocaine 15 mg/kg, black circles (n = 4).

In group 3, i.e. in rats that received pre-treatment with the D3 antagonist SB-277011-A (10 mg/kg i.p.) followed by acute cocaine administration (15 mg/kg i.p.) showed larger increase in both areas of the DA levels than that monitored after cocaine alone, as shown in Figure 4. In particular the figure shows the last 15 min recordings of the first treatment period (i.e. vehicle for the group 1 vehicle + vehicle, vehicle for the group 2 vehicle + cocaine or SB-277011-A for the group 3 SB-277011-A + cocaine, respectively) that have been used as baseline. Furthermore, since no significant difference in DA release was evaluated between shell and core when comparing the effect of cocaine in both areas (group 2), the effects occurred in each area were combined as single entity. Thus these values were plotted for comparison versus vehicle (group 1) and versus SB-277011-A + cocaine (group 3) treated rats. Briefly, they reached approx. 280 % in shell and 310 % in core relating to basal levels, respectively. Precisely, the three way ANOVA showed a significant effect of treatment F_(2,25)_ = 5.14, p = 0.013, time F_(12,30)_ = 5.65, p = 0.001 and treatment by time interaction F_(24,30)_ = 10,62, p = 0.001 (Figure 4). Finally, planned comparison *post-hoc* test demonstrated a significant difference between cocaine alone (group 2) and SB-277011-A + cocaine (group 3) at times 80 — 95 min.

#### 3.1.1. Basal DA levels, comparison with the literature

A number of reports indicate differences in shell and core DA metabolism [8, 9], DA reuptake [41], pattern of synaptic [42] and behavioural responses [43, 44] elicited by direct application of DA analogues. Besides, previous *in vivo* microdialysis results have reported either no significant differences in core and shell extracellular DA levels [45 - 48], or higher DA extracellular levels in core than in shell [49, 50] or, conversely higher basal DA levels in shell than in core [8, 51]. Consequently, shell / core differences in basal DA levels constitute currently a matter of debate [15]. In this sense, Frank et al., 2008 performed a meta-analysis on published data from 116 experiments out of 266 publications obtained using in vivo microdialysis in NAc [52]. In this report no apparent differences in basal DA levels between shell and core in rats [i.e 4.15nM and 2.95nM, respectively] was revealed. Accordingly, the mean basal levels monitored in the present work [see above] are not different from those reported in Frank *et al.* [52], as well as from those reported in an earlier voltammetric study [53].

However, another voltammetric study proposed considerably higher concentration of basal DA levels in NAccore, i.e. in the micromolar range [54]. The different type of electrodes used (i.e not coated with Nafion [32] or nacro [33] that allow to distinguish between biogenic amines and respective metabolites i.e. DA and DOPAC, nanomolar or micromolar concentrated within the extracellular fluid, respectively) and the different electrochemical method used (i.e fast-scan cyclic voltammetry) may explain such difference in basal DA concentrations. Again, a recent voltammetric work showed a short lasting (seconds) increase of DA-related signal in shell but not in core that was detected in rats given free-choice access to a novel environment. This increase occurred only during the initial entry into the novel compartment and has been discussed by the authors as possibly related to a mixture of DA, DOPAC, NA as the very fast voltammetric method used with sensors without nafion coating is unable to differentiate between these chemicals having similar oxidation potentials [16]. On the other hand, a more recent report using a slower voltammetric method together with nafion treated sensors indicates that DA neurons innervating the core or the shell appear to be involved specifically in the response to the conditioning to an aversive or salient olfactory stimulus, respectively although each individual pathway responded following a similar pattern [17].

#### 3.1.2. Effect of cocaine on basal DA levels

The present work also shows that acute treatment with 15 mg/kg ip cocaine resulted in a similar increase in extracellular DA levels in both core and shell. Again, this result is in accord with the above mentioned meta -analysis study [52] indicating 10-15 mg/kg as optimal dose of cocaine given ip (or iv) in rats (and mice) and that at these dosages no significant differences in dopaminergic response between NAccore and NAcshell were observed relatively to baseline. Only at higher cocaine dosage was the slope of a dose-response curve bigger in shell than in core.

Furthermore, these data confirm the effective selectiveness of the Nafion biosensors used as it is known that while cocaine is increasing DA levels in brain areas, it also produced a significant decrease in dihydroxyphenylacetic acid (DOPAC) levels [55, 56]. Therefore, taken together with in vitro tests showing that the Nafion mCFE appeared to be insensitive to DOPAC at micromolar concentration [32], these *in vivo* findings confirm that the electrochemical signal measured in this study is due to the oxidation of DA and not of DOPAC.

Previous works have shown that DA concentrations were high in the nAc, and that noradrenaline (NA) and serotonin (5-HT) concentrations were considerably lower than the DA concentration [57]. In particular, HPLC studies with ECD detection and direct collection of the peaks measured by liquid scintillation spectrometry showed that DA levels in the nAccumbens are nearly 900 pmol/mg protein versus NA levels of approximately 19 pmol/mg protein [58]. In addition, in vitro analysis using DPVoltammetry showed that the Nafion mCFE has similar sensitivity to DA and NA. Therefore since the NA content in nAc is in the order of 2.1% of the whole catecholamines, we consider the in vivo voltammetric signal monitored in the nAc as mainly related to DA level.

#### 3.1.3. Effect of SB27701l-A treatment

The dopamine D3 receptor is mainly expressed in regions of the brain associated with the limbic system and in particular in the shell of the NAc [59-61]. These receptors have been implicated in the modulation of cocaine-related behaviours as SB-277011-A reduces cocaine-induced electrical brain stimulation and conditioned place preference [25] as well as cocaine-seeking behaviours [29, 40]. It has been suggested that stimulation of NAc D3 receptors localized on the postsynaptic membrane are involved in cocaine reinforcement [62]. However, recent findings indicate that these receptors are not involved in the direct reinforcing action of drugs of abuse, while they appear to modulate, i.e. to decrease the motivation to take drug in a way that is proportional to the “price of the drug” [63] i.e. FR schedule with a relatively high response – requirement (FR 10) in cocaine self-administering rats [64]. In our hands**,** voltammetric DA levels measured in both core and shell subregions were not significantly affected by treatment with SB-277011-A. However, pre-treatment with SB-277011-A induced a significant boost of the effect of a subsequent systemic cocaine administration in respect to the effects of cocaine alone (figure 2). This indicates that SB-277011-A could modulate the efficacy of a drug of abuse such as cocaine. Similar effects were observed for amphetamine in experiments using *in vivo* fMRI. Indeed, the amphetamine-induced activity measured by the rapid relative cerebral blood volume (rCBV) in the NAc was enhanced by SB-277011-A pre-treatment while the compound alone produces only limited changes in rCBV [65].

Although PK direct interaction between cocaine and SB-277011-A can not be excluded the augmented hemodynamic response reported [65] is consistent with the increased extracellular DA upon cocaine challenge observed in the nAcc following dopamine D3 receptor antagonism by SB-277011-A in naive rats [66]. On the other hand, the enhancement of the amphetamine response observed in the above fMRI study is in contrast to the attenuation in rCBV response that has been observed when pre-treating with DA Dl receptor antagonists [67].

Altogether, these data suggest that the dopamine D3 receptor might mediate an inhibitory action on downstream activity in limbic circuits and that blockade of such receptor by SB-277011-A may enhance the effect of cocaine i.e. via increased extracellular DA concentration in a number of brain regions as reported by Schwarz *et al.* [68]. This may occur either through the inhibition of the DA D3 autoreceptors (that regulate DA synthesis or release presynaptically [69]) or/and via attenuation of feedback loop inhibition as SB-277011-A may act postsynaptically by jamming the inhibitory action of D3 receptors on neuronal activity mediated by dopamine D1 receptors. A further hypothesis may consider a putative effect of D3 receptors regulating DAT. However this possibility could be ruled out as no changes in extracellular DA levels were monitored following treatment with SB-277011-A alone.

All these observations concur with the present evaluation of a modulating influence of D3 receptor antagonism on the cocaine effect on DA activity in the nAc.

## 4. Conclusions

This work demonstrates that this new dual-probe mCFE approach can be used to monitor extracellular levels of DA in discrete brain regions as reduced in dimension and interconnected as core and shell of NAc. Further, this technique can discriminate between discrete neurochemical changes occurring in response to pharmacological challenge(s).

Main evidence of the present work is that acute treatment with 15 mg/kg i.p. cocaine resulted in a similar increase of extracellular DA levels in both core and shell. This data is in accord with the most recent meta-analysis on in vivo microdialysis studies on cocaine–induced DA overflow in the NAc [52]. Another main result is that the D3 receptor antagonist SB-277011-A alone is not affecting DA outflow while it modulates the effects of cocaine on DA, allowing one to suggest that the reported drug of abuse-related behavioural effects of SB-277011-A might be due to the ability of the compound to further enhance the extracellular effects of DA in the NAc when it is administered before abused chemicals such as cocaine or amphetamine. As a result, in such conditions the effect on the dopaminergic system could be obtained with a lesser amount of drug of abuse.

This interpretation agrees with the recent suggestion that the dopamine D3 receptors are more implicated in the motivation to self-administer drugs of abuse than in their direct reinforcing effects [70] and with the proposed activity of the D3 receptor antagonist in normalising DA release in the condition of reduced DA release as recently reported in drug dependent people [71].

Finally, these data, together with the reported efficacy of such a compound on attenuating reinstatement of drug-seeking behaviour [18] leads one to suggest that SB-277011-A may be useful within the treatment of drug addiction and in particular to prevent craving for drugs of abuse.
